# The paracrine signalling between endothelial and pancreatic β-cells: implications for oxidative stress and diabetes

**DOI:** 10.1007/s40618-026-02869-5

**Published:** 2026-03-25

**Authors:** Adrián Idoate-Bayón, Elena Ainzúa, Javier Marqués

**Affiliations:** 1 Diabetes Cure Research Association (DCRA), Villava, Navarra, Spain; 2 Department of Biochemistry and Genetics, University of Navarra, Pamplona, Spain; 3 Navarra Institute for Health Research (IdiSNA), Pamplona, Spain; 4 Faculty of Medicine and Dentistry, William Harvey Research Institute, Barts & The London Hospitals, Queen Mary University of London, Charterhouse Square, London, UK

**Keywords:** Endothelial cells, Pancreatic β-cells, Oxidative stress, Diabetes mellitus, Paracrine signalling, GSIS

## Abstract

**Background:**

The Langerhans islets are highly vascularized structures that regulate plasma glucose levels. These islets are primarily composed of different types of endocrine cells (α, β, δ, PP, and ε) and endothelial cells. The paracrine signalling between pancreatic β-cells and endothelial cells is key for maintaining β-cell mass and preserving endocrine function. Oxidative stress, caused by an excess of reactive oxygen species (ROS), is a pathological mechanism that damages pancreatic β-cells and endothelial cells.

**Aim:**

In this review, we discuss how oxidative stress impacts not only on endothelial and pancreatic β-cells individually, but also on their paracrine signalling.

**Results:**

Oxidative stress triggers apoptosis and dysfunction of endothelial and pancreatic β-cells, as well as it alters glucose-stimulated insulin secretion (GSIS) from pancreatic β-cells. Moreover, oxidative stress affects to the homeostasis of vascular endothelial growth factor (VEGF) and Heparin-binding Epidermal Growth Factor (HB-EGF), two key mediators of the β-cell-endothelial cell crosstalk. Finally, an oxidative environment in the Langerhans islets promotes inflammation by increasing the secretion of cytokines, which impact in both cell types, further impairing insulin production.

**Conclusion:**

These effects of oxidative stress on the endothelial cell-β-cell paracrine signalling may represent a promising therapeutic target to alleviate diabetes development and its derived complications.

## Introduction

### Diabetes mellitus

Diabetes mellitus (DM) is a chronic metabolic disease characterized by abnormally elevated blood glucose levels. Type 1 diabetes (T1D) and type 2 diabetes (T2D) represent the two main subtypes of this pathology. T1D is the consequence of an autoimmune attack against pancreatic β-cells. These cells are present in the islets of Langerhans, and their main function is the production of insulin, a hormone with hypoglycaemic action [1]. Patients suffering from T1D require daily administration of insulin. The onset of T1D mainly occurs at childhood/adolescence, although the number of cases identified in adulthood have increased recently [2]. T1D accounts for 5–10% of total diabetes cases worldwide presenting geographic variations in incidence [3]. The current incidence rates per 100,000 inhabitants of T1D in some of the developed countries are: 32.7 in Norway [4], 22–24 in the USA [5], 13–18 in Spain [6, 7], 2–5 in China, Japan, Mexico and India [8–11].

T2D has a multifactorial origin, and it is primarily characterized by an imbalance between insulin demand and supply. There are two possible mechanisms causing T2D: a peripheral insulin resistance (IR), or an inadequate insulin secretion by β-cells in response to glucose [1]. A wide range of therapeutic strategies is available for T2D patients, spanning from lifestyle modifications to diverse pharmacological interventions including metformin, sodium-glucose co-transporter 2 (SGLT2) inhibitors, sulfonylureas, and Glucagon-Like Peptide-1 (GLP-1) agonists, among others [12]. T2D typically debuts later in life, although its prevalence among the younger populations is increasing [13]. Globally, T2D accounts for approximately 90–95% of all diabetes cases with homogenous prevalence between different countries. For instance, prevalence rates are estimated at 14% in the United States and Spain [14], 12.8% in China [15], 11.4% in India [16] and 9.5% in Mexico [17].

Both T1D and T2D are associated with a significant reduction in both quality of life and life expectancy. In the case of T1D, life expectancy is estimated to be 11–18 years shorter when compared to the general population [18, 19]. In addition, patients experience significant impairment in their quality of life due to both, direct and indirect complications. Direct complications include hypoglycaemia, hyperglycaemia, and diabetic ketoacidosis, which can lead to life-threatening episodes requiring immediate management. Indirect complications, largely driven by endothelial dysfunction, include diabetic retinopathy, neuropathy, myocardial infarction, and arterial hypertension, among others [20]. Importantly, diabetes also exerts a considerable burden on mental health, with patients frequently experiencing depression, anxiety, and stress [21].

Altogether, these considerations underscore the urgent need for continued research into diabetes pathophysiology, focusing on its prevention and treatment, with the aim of halting disease progression and mitigating its consequences.

### Structure and function of the endocrine pancreas

The pancreas is an elongated gland anatomically divided into head, body, and tail. The pancreas comprises an exocrine and an endocrine component. The exocrine pancreas accounts for approximately 80% of the total organ mass and consists of acinar units, which synthesize pancreatic juice for digestion; and a ductal system, which conveys these secretions into the duodenal lumen. Then, approximately 18% of the pancreas is composed of stroma, vasculature, and innervation [22]. Finally, the endocrine pancreas constitutes the remaining 1–2% of the total mass and it is spread through the organ as clusters of cells known as islets of Langerhans [23]. The principal function of these islets is the regulation of blood glucose levels. Five major endocrine cell types are found within the islets. β-cells comprise 60–70% of islet cells and secrete insulin; α cells account for 15–20% and secrete glucagon; and the remaining minor populations (δ, PP, and ε cells) represent < 10–15% and secrete somatostatin, pancreatic polypeptide, and ghrelin, respectively [24]. The islets of Langerhans are highly vascularized by a dense capillary network. A sufficient islet perfusion is crucial for the adequate cellular function and for accurate, moment-to-moment regulation of glycaemia [25].

### Cellular components of the endocrine pancreas: β-cells and endothelial cells

#### Pancreatic β-cells

Pancreatic β-cells play a fundamental role in systemic glucose regulation, as it is the only cell type capable of synthesizing insulin. Beyond its hypoglycaemic action, insulin also exerts lipogenic effects, promotes amino acid synthesis and stimulates cell growth and survival [26]. Nevertheless, β-cells exert functions beyond insulin secretion. C-peptide, which is released into the bloodstream in equimolar amounts with insulin, has been reported to exert beneficial effects on the microcirculation, neuronal function [27], and sexual capacity [28], as well as protection against osteoporosis [29]. Amylin is another hormone exclusively synthesized by pancreatic β-cells. The physiological functions of amylin include the delay of gastric emptying [30], the inhibition of glucagon secretion [31], and early satiety [32]. Amylin aggregation into amyloid deposits has been related to β-cell death and the pathogenesis of T2D [33]. Another molecule exclusively expressed by β-cells is urocortin 3 (UCN3), which stimulates δ-cells, promoting somatostatin release, which in turn inhibits insulin and glucagon secretion [34]. Interestingly, UCN3 has been proposed as a reliable marker of β-cell maturation [35].

The process of insulin synthesis begins with the expression of the *INS* gene, regulated by the transcription factor Pancreatic and Duodenal Homeobox 1 (PDX-1), a stablished marker of the β-cell lineage. Lack of PDX-1 abrogates the compensatory response of β-cells to IR, impairs glucose homeostasis, and may contribute to the pathogenesis of T2D [36]. Insulin is synthesized as a pre-pro-peptide and processed to proinsulin by signal peptidases. Proinsulin is then converted by prohormone convertases and carboxypeptidases into insulin and C-peptide, which are stored in secretary granules and released on demand [37]. β-cells possess a sensing mechanism that enables them to monitor plasma glucose levels. To sense the nutritional state, β-cells are organized into islets that are strategically connected to the vasculature. Interestingly, although the islets only comprise 1–2% of the pancreas, they receive up to 20% of the pancreatic blood supply [38]. The sensing mechanism relays on the glucose transporter 2 (GLUT-2). When its plasma levels increase, glucose enters β-cells through GLUT-2 and undergoes complete oxidation via glycolysis and Krebs cycle, resulting in an increased ATP/ADP ratio. This rise in ATP leads to the closure of ATP-sensitive potassium channels (K_ATP_). The subsequent membrane depolarization opens voltage-dependent calcium channels, allowing calcium influx and ultimately triggering the exocytosis of insulin-containing granules [39].

In addition to glucose, paracrine and endocrine signals finely modulate β-cell insulin secretion, which highlights the relevant connection between these cells, the neighbouring cells and the vasculature. Within the islets of Langerhans, glucagon secreted from α-cells exerts a context-dependent effect, amplifying insulin release at moderate glucose levels [40]. Somatostatin produced by δ-cells acts as a potent inhibitor of insulin release through activation of somatostatin receptor subtype 2 on β-cells [41]. Conversely, hormones such as GLP-1 and glucose-dependent insulinotropic polypeptide potentiate insulin secretion by enhancing cAMP production and amplifying calcium-dependent exocytosis [42]. These regulatory inputs highlight the integration of nutrient, hormonal, and neuronal components that determine the magnitude and timing of insulin release into the circulation.

#### Endothelial cells

Endothelial cells are present in every blood vessel of the organism. They constitute a single monolayer of cells (the endothelium) in direct contact with the blood. These cells are key regulators of vascular homeostasis, including blood vessel tone, vascular permeability, and leukocyte trafficking. In addition, under physiological conditions, the endothelium presents a crucial antioxidant, anti-inflammatory, and antithrombotic phenotype [43]. At the pancreas, endothelial cells create a specialized vascular network within islets. In this environment, endothelial cells and β-cell maintain a paracrine communication, which consists in different signalling pathways in both directions that support β-cell homeostasis and insulin secretion.

These specialized endothelial cells from the pancreas are fenestrated, a configuration created in response to vascular endothelial growth factor-A (VEGF-A) and angiopoietin-1 secreted by islet cells. In diabetes, this specialized vascular architecture becomes compromised, with islet capillaries exhibiting thickening, dilatation, and fragmentation, while endothelial cells express markers of inflammation and activation. In vitro studies indicate that this dysfunctional endothelial phenotype directly contributes to impaired insulin secretion from β-cells [44–46]. To sum up, pancreatic endothelial cells mediate essential paracrine signals crucial for β-cell proliferation, differentiation, and pancreatic islet function.

## The crosstalk between pancreatic β-cells and endothelial cells in diabetes

### Pancreatic β-cells in diabetes

The pancreatic β-cell is the central pathological player in both major forms of diabetes. In T1D, pancreatic β-cells are attacked by the immune system. In this case, genetic predisposition might be involved, particularly through alterations in the major histocompatibility complex (HLA). In T1D patients specific class II haplotypes confer the highest risk by affecting the autoantibodies firstly produced by the patient [47]. However, genetics alone cannot fully explain the onset of T1D diabetes; environmental factors also play a pivotal role in triggering the disease. Among these factors, viral infections have been proposed as key initiators of β-cell autoimmunity [48]. At the onset of the autoimmune attack, resident macrophages within the islets of Langerhans undergo a phenotypic shift from an anti-inflammatory to a pro-inflammatory state. This polarization enables the secretion of cytokines, both locally and systemically, which facilitate the recruitment of the first dendritic cells [49]. Dendritic cells within the islets of Langerhans capture β-cell antigens and present them in the pancreatic lymph nodes, where autoreactive CD8⁺ T lymphocytes clonally expand. In parallel, B lymphocytes become activated and produce autoantibodies against these antigens, thereby accelerating the autoimmune response [50]. This process progresses through the recruitment and infiltration of additional immune cells, ultimately leading to the destruction of pancreatic β-cells [51]. Some of the autoantibodies against β-cell produced by B lymphocyte act against insulin, glutamic acid decarboxylase, the tyrosine phosphatase IA-2, and the zinc transporter (ZnT8) which accelerates β-cell destruction [52].

There are different origins of T2D, with up to five subtypes described [53]. The most prevalent cause of T2D is IR in peripheral tissues. Obesity and overweight strongly correlate with increased IR. As fat tissue accumulates in different organs, it causes lipotoxicity, which can lead to T2D due to β-cell dysfunction and apoptosis [54]. During obesity, cholesterol and fatty acids travel from mature adipocytes to ectopic sites causing lipotoxicity and systemic IR [55]. Moreover, the inflamed white adipose tissue of obese patients increases the release of IL-1β, TNF-α and IL-6, which favours IR [56]. Furthermore, an increase in non-esterified fatty acid (NEFA) causes IR and restricts insulin secretion by β-cells in the pancreas [57]. NEFA metabolism also produces toxic metabolites, such as diacylglycerol and ceramides, that provoke IR through the impairment of insulin signalling [58].

### Endothelial cells in diabetes

The disruption of endothelial function contributes significantly to the pathogenesis of both microvascular and macrovascular complications of diabetes. During diabetes, endothelial dysfunction is triggered by chronic hyperglycaemia, IR, and the associated metabolic disturbances. Exposed to these chronic pathological conditions, endothelial cells decrease nitric oxide (NO) biosynthesis, accompanied by increased inflammation, endothelial-mesenchymal transition, cell senescence, and cell death. These alterations result in impaired vasodilatation, enhanced adhesion molecule expression and the remodelling of the extracellular matrix. Collectively, the maladaptation of endothelial cells contributes to vascular complications [59, 60]. Macrovascular complications include atherosclerosis-related conditions as coronary artery disease, cerebrovascular disease or peripheral artery disease. Microvascular complications are characterized by basement membrane thickening, and include diabetic nephropathy, retinopathy, neuropathy, and cardiomyopathy [60, 61].

Beyond their conventional vascular regulatory functions, endothelial cells in diabetes exhibit compromised nutrient and hormone transport to peripheral tissues. Under physiological conditions, endothelial cells facilitate glucose and insulin exchange through insulin-independent transport mechanisms. Nonetheless, the dysfunctional phenotype induced at diabetic conditions compromises the capacity of endothelial cells to distribute glucose. In a positive feedback loop, the impaired tissue perfusion and substrate delivery of endothelial cells during diabetes exacerbates systemic IR. Therefore, endothelial dysfunction represents a pathophysiological link between vascular complications and metabolic dysregulation in diabetes [62, 63].

In T2D, the alterations of the vascular network of the pancreas are different across animal species. In human diabetic islets, capillaries present an abnormal morphology with increased thickening and fragmentation, which is accompanied by fibrosis. In murine animal models of diabetes, in addition to these abnormalities, the pancreatic tissue may also present oedema and haemorrhage [64, 65]. The pancreatic endothelial cells display functional changes in diabetic conditions like other endothelial cells. More specifically, these endothelial cells present VEGF-A desensitization and limited insulin outflow into systemic circulation, which has a direct impact on glucose levels management [66, 67]. Due to its different aetiology, T1D produces different effects in the vascular network of the pancreas. As an example, there is an increased vascular density and lymphocyte adhesion to the endothelium. The transendothelial migration of immune cells to the pancreas tissue contributes to the autoimmune β-cell destruction and disease pathogenesis [68].

### Endothelial-β cell crosstalk in physiological and diabetic conditions

As previously stated, the pancreas is a highly vascularized organ, which is an essential feature for its endocrine function. Under both, homeostatic and diabetic conditions, endothelial cells and pancreatic β-cells maintain close interaction.

The experimental evidence supporting this crosstalk has been obtained mainly from in vitro and in vivo models. For instance, co-culture of MIN6 (β-cells) and MS1 (endothelial cells) within a hydrogel recapitulates an islet-like pattern of cellular organization. This combination of cells exhibits a higher glucose stimulated insulin secretion (GSIS) compared to MIN6 cells cultured alone [69]. A similar model combining INS1E cells and HUVECs in a collagen type 1 matrix promotes the expression of extracellular matrix proteins such as Laminin, nidogen-1 (Nid1) and Collagen IV. HUVECs also protect INS1E cells from hypoxic stress [70]. Although these results provide mechanistic insights into endothelial cells effects on β-cells, the coculture models combining immortalized cell lines and simple matrices may not fully recapitulate the complexity of the pancreatic tissue. Some in vivo evidence also seems to support this mechanism, as the injection of bone marrow-derived endothelial cells to NOD mice increased β-cell proliferation [71].

This beneficial interaction between endothelial and pancreatic β-cells is potentially mediated by soluble factors and adhesion proteins. Among the soluble factors, VEGF-A produced by pancreatic β-cells is recognized as a key player in this paracrine signalling; however, there is some debate about its role. On the one hand, VEGF-A is considered a critical driver of the revascularization of the endocrine pancreas, with special relevance during development [72]. Some studies using murine ex vivo cultures show that VEGF-A induces hepatocyte growth factor (HGF) production in endothelial cells, which in turn stimulates β-cell proliferation [73]. Consistent with this, in vivo studies showed that hypoglycaemic conditions reduce the secretion of VEGF-A, which leads to decreased vascularization of the pancreatic islets. Interestingly, the supplementation with VEGF-A prevented apoptosis of endothelial and pancreatic β-cells, maintaining the β-cell mass in hypoglycaemic mice [74]. On the other hand, there is evidence indicating that VEGF-A effects may depend on the context. In vivo experiments in normoglycemic mice demonstrated that VEGF-A overproduction is associated with reduced β-cell mass, increased vascularization, and infiltration of bone marrow-derived macrophages [75]. Similar reports suggest a developmental role for VEGF-A during embryogenesis, while an excess of VEGF-A under diabetic conditions can increase inflammation and aggravate the disease [76, 77]. Other members of the VEGF family, such as VEGF-B, may also be involved in insulin production from pancreatic β-cells [78, 79]. Overall, current data suggest a dual and context-dependent role for VEGF-A in this paracrine signalling: physiological levels of VEGF-A are essential for vascularization and β-cell homeostasis, whereas an overproduction could lead to inflammation and, finally, β-cell dysfunction. These data are derived from in vitro systems or murine in vivo models, however the exact mechanism that VEGF-A plays in this paracellular crosstalk in diabetic patients remains to be addressed.

Different studies using co-cultures of immortalized cell lines and murine islets identified soluble factors that may contribute to the paracrine communication between endothelial and pancreatic β-cells. For instance, in murine islets and INS-1 cells, triose-phosphate-isomerase (TPI) produced by endothelial cells reduces GSIS [80]. On the contrary, nitric oxide (NO) produced by EA.hy929 (endothelial) cells induces insulin secretion of INS-1 cells when co-cultured [81]. Beyond insulin secretion, effects on the differentiation of β-cells were also observed. The co-culture of mouse embryonic bodies with human microvascular endothelial cells results in higher differentiation levels compared to the co-culture with fibroblasts. These embryonic bodies exhibited higher PDX-1, insulin-1 and insulin-2 expression levels [82]. These findings are derived exclusively from in vitro and ex vivo systems. Although these data help to understand the interaction between endothelial and β-cells, whether these mechanisms have a correlate in human islets or in diabetes remains unknown.

Secreted T-cadherin is another relevant soluble factor mediating the communication between endothelial and pancreatic β-cells. Interestingly, insulin reduces the secretion of T-cadherin from HUVECs. This interaction becomes especially relevant in diabetic conditions, as endothelial-specific T-cadherin *knock-out* mice exhibit higher glucose levels after streptozotocin administration [83]. Similarly, global T-cadherin *knock-out* mice subjected to streptozotocin have an impaired glucose metabolism and reduced β-cell mass in response to high fat diet. Subsequent supplementation with T-cadherin reverses the pathological phenotype [84]. Other soluble factors, including fibroblast growth factor-2 (FGF-2), hepatocyte growth factor (HGF), Pdk1, endostatin and Serpin E1, have also been linked to the communication between endothelial cell and pancreatic β-cells [85–87]. These reports of different in vivo and in vitro studies demonstrating a paracrine signalling by soluble factors indicate the need of human studies that may confirm their correlate at physiological and diabetic conditions.

Protein-mediated cell-to-cell contact also contributes to the paracrine interactions between these cell types. For instance, in a co-culture of NIT-1 or βTC3 cells together with MS1endothelial cells, the endothelial cells can glycosylate and promote the maturation of integrin-β1 expressed in pancreatic β-cells, increasing glucose sensitivity and insulin gene expression [88]. Nonetheless, these results are limited to in vitro cocultures. Moreover, in vivo studies show that the interaction between the EGF receptor from β-cells with HB-EGF from endothelial cells induces cell cycle progression in β-cells and the physiological development of the pancreas [89]. Finally, leucine-rich α2-glycoprotein 1 is overexpressed in pancreatic tumours promoting the proliferation of β-cells in vitro. However, this protein is also involved in tubule formation by HUVECs, suggesting a role in angiogenesis [90]. Again, scarce information on these cell-to-cell contacts is available in human tissue, which may or may not reflect the findings of these reports.

Recently, some authors have suggested how bone marrow and its derived cells can play a key role in this system. On the one side, in mice, endothelial cells derived from the bone marrow migrate to the pancreatic islets after streptozotocin injection. This indicates that endothelial cells mediating β-cell recovery may originate from outside the pancreatic tissue [91]. On the other side, a co-culture of endothelial, β-cells and macrophages derived from human pluripotent stem cells enhances endothelial and β-cell markers. Interestingly, when these cells are subjected to a viral infection, macrophages triggered pyroptosis of pancreatic β-cells [92].

**Fig. 1:**
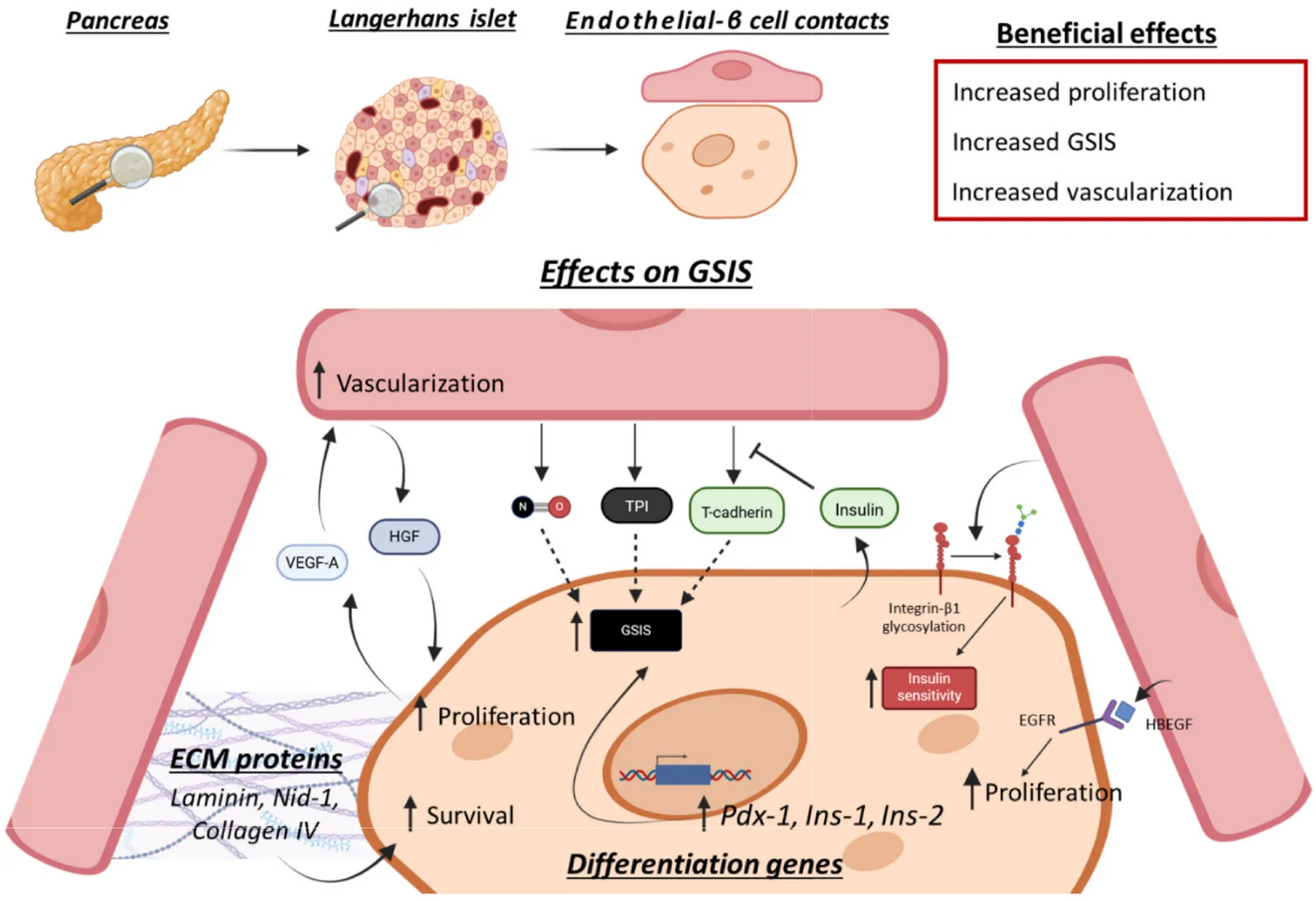
Main paracellular communication pathways between endothelial cells and pancreatic β-cells under physiological conditions. Under *physiological conditions*, the signalling pathways between endothelial and pancreatic β-cells lead to four main effects: (i) *β***-***cell proliferation* (HGF, HBEGF) and islet *vascularisation* (VEGF-A); (ii) *β-cell viability* by increasing extracellular matrix deposition (Laminin, Nid-1, Collagen IV); (iii) expression of *differentiation* genes in pancreatic β-cells (Pdx-1, Ins-1, Ins-2); and (iv) *improved glucose-induced insulin secretion* (NO, TPI, T-cadherin) and *insulin sensitivity* (Integrin-β1). EGFR: epidermal growth factor receptor. HBEGF: heparin-binding epidermal growth factor-like growth factor. HGF: hepatocyte growth factor. Ins-1: insulin (1) Ins-2: insulin (2) Nid-1: nidogen-1. NO: nitric oxide. Pdx1: pancreas/duodenum homeobox protein 1. TPI: triose phosphate isomerase. VEGF-A: vascular endothelial growth factor A. Figure created using Biorender

The present section highlights the increasing interest in the endothelial cell-pancreatic β-cell communication (Fig. 1). The interaction between endothelial cells and β-cells results in different beneficial effects. Firstly, NO, TPI and T-cadherin produced from endothelial cells regulate the GSIS of β-cells [80–81, 83]. The coculture of these cell types promotes the expression of β-cell markers (Pdx-1, Ins-1, Ins-2), increasing the GSIS [82]. Secondly, Integrin-β1 glycosylation of β-cells by endothelial cells increases insulin sensitivity. Thirdly, HBEGF and HGF produced by endothelial cells induce β-cell proliferation [85–87]. Finally, the increased synthesis of ECM proteins induced by the coculture of these cells promotes cell survival. Nonetheless, the molecular factors involved in modulating this interaction during the progression of diabetes require further investigation, as the vast majority of these findings were obtained from in vitro studies of individual cell lines or cocultures. Therefore, there is an urgent need to demonstrate these mechanisms in vivo and at human tissue to elucidate their actual relevance in human diabetes.

## Oxidative stress in diabetes mellitus

### Sources of oxidative stress

One of the pathological mechanisms that damages endothelial and pancreatic β-cells is oxidative stress. Oxidative stress is the pathological consequence of an accumulation of reactive oxygen species (ROS). ROS are derived from O_2_-related metabolism and chemical reactions. This group of molecules is mainly composed of superoxide (O_2_^•−^), hydrogen peroxide (H_2_O_2_), hydroxyl radical (HO^•^), and peroxynitrite (ONOO^−^) [93]. ROS play key physiological signalling roles. For instance, ROS can regulate the vascular tone by reducing the bioavailability of NO [94]. However, when the delicate balance between the production and destruction of ROS is disrupted, oxidative stress arises. This cellular mechanism triggers the unfolded protein response, proinflammatory signalling pathways, or even cell death by apoptosis [93].

There are different enzymatic sources of ROS in the organism, mainly generated as a subproduct of enzymatic reactions. Xanthine Oxidase (XO) and the Cytochrome P450, lead to the generation of ROS as a subproduct of uric acid production and xenobiotic clearance. NO synthase (NOS) produces O_2_^•-^ in the absence of essential cofactors such as BH4. The electron transport chain leaks electrons that react with O_2_, generating O_2_^•‐^ [95]. Finally, another relevant enzymatic source of ROS is the family of the NADPH oxidases (NOX). This family is divided into seven different members: five NOX homologs (NOX1-5), and two dual oxidases (DUOX1-2). The products of these enzymes are: O_2_^•‐^, in the case of NOX1, NOX2, and NOX5; and H_2_O_2_ in the case of NOX4, DUOX1 and DUOX2 [96, 97].

Oxidative stress, generated from these sources, is an underlying mechanism causing endothelial dysfunction and damaging pancreatic β-cells. This is especially relevant in diabetes, where these cell types play a key role [98]. A detailed description of how oxidative stress affects endothelial and pancreatic β-cells is essential for the understanding of this metabolic disease.

### Oxidative stress in endothelial cells

Elevated glucose levels and altered metabolic processes enhance ROS generation in the pancreas. The oxidative environment affects not only β-cells, but also the endothelial cells within the islet microvasculature [99, 100]. Oxidative stress synergistically compromises microvascular blood flow and O_2_/nutrient exchange, accelerating islet deterioration, endothelial dysfunction, and contributes to the metabolic and vascular IR [101, 102]. Human studies show that hypoglycaemia and glucose variability (common in T1D) increase cardiovascular risk by causing endothelial dysfunction and stimulating the secretion of proinflammatory cytokines such as IL-6 and TNF-α, creating a vicious cycle of oxidative stress and vascular inflammation [103, 104]. This proinflammatory environment can have a direct impact in pancreatic β-cells at the Langerhans islets, the impairment of β-cell function.

In mice, endothelial cells in T1D exhibit epidermal growth factor receptor (EGFR) overactivation as well as its downstream oxidative cascade. In fact, the inhibition of this cascade partially reverses endothelial dysfunction [105]. In parallel, in a study performed with rats, antioxidant components counteract hyperglycaemia-induced mitochondrial dysfunction by strengthening endogenous antioxidant defences through upregulation of protective enzymes such as SOD2 and hemooxygenase-1 [106]. In T2D chronic hyperglycaemia drives increased ROS production in endothelial cells through multiple pathways, including mitochondrial electron transport chain leakage, the activation of NADPH oxidase, the formation of advanced glycation end products (AGEs), or protein kinase C (PKC) activation [107].

Several therapeutic interventions that target oxidative stress have demonstrated significant protective effects on endothelial function in T2D. These treatments act through multiple complementary mechanisms, including the inhibition of the PKC-β/NOX axis and inflammatory cascades involving NF-κB/iNOS. Additionally, the reduction in mitochondrial ROS production from immunoinflammatory cells contributes to improved endothelial function in diabetic patients. Collectively, these findings derived from in vivo and human studies, strongly suggest that pharmacological strategies aimed at mitigating oxidative stress represent a promising therapeutic approach for preserving endothelial integrity and preventing vascular complications in T2D [108–110].

Interestingly, two different studies have demonstrated how intermittent high levels of glucose lead to ROS production in endothelial cells. This situation causes cell senescence and seems to be linked to NOX activity in porcine and human endothelial cells [111, 112]. Additional studies show that the activation of NOXs correlates highly with endothelial dysfunction in a porcine model and in human endothelial cells, via NOX2 and NOX5 respectively [113, 114]. This relationship between fluctuating glucose levels and ROS production, although potentially relevant, has been scarcely studied and could potentially cause pancreatic β-cell dysfunction.

Oxidative stress also triggers an inflammatory response in endothelial cells. In vitro, NOX5 activity increases the expression of intracellular adhesion molecule 1 (ICAM-1) and vascular cell adhesion molecule 1 (VCAM-1) in HUVECs [115]. Interestingly, NOX5 has been demonstrated to be directly activated by glucose [116]. Moreover, NOX4 promotes the expression of ICAM-1 and VCAM-1 in response to TNF-α stimulation in HUVECs [117]. NOX4 also potentiates the secretion of IL-8 and monocyte chemoattractant protein 1 (MCP-1) in human endothelial cells in response to toll-like receptor 4 (TLR-4) activation [118, 119]. Similarly, palmitate induces IL-6 secretion through the activation of the TLR-4/NOX4 crosstalk [120]. A similar pathway is observed in endothelial cells from diabetic Akita mice, where NOX5 is activated by the TLR-4 receptor and leads to the secretion of MCP-1 [121]. NOX5 activity also activates cyclooxygenase-2 and prostaglandin E_2_ (PGE_2_) secretion in human endothelial cells [122]. Again, these proinflammatory mediators are locally released, which means that oxidative stress in endothelial cells could have a final effect on pancreatic β-cells at the Langerhans islet. Nevertheless, this potential pathological mechanism has yet to be fully addressed.

This section describes extensive in vitro, in vivo and human studies which show how oxidative stress can lead to different effects in endothelial cells such as endothelial dysfunction, apoptosis, adhesion molecule expression, and the secretion of proinflammatory mediators. This results in a proinflammatory environment that may lead to the infiltration of immune cells into the tissue. In the case of the pancreatic tissue, this immune cell infiltration may trigger the onset and/or accelerate the consequences of diabetes. Additionally, oxidative stress originated in endothelial cells could affect β-cell function and viability, a topic addressed in the next section.

### Oxidative stress in pancreatic β-cells

Oxidative stress was described decades ago as a pathological mechanism in pancreatic β-cell lines [123]. Interestingly, the transcription factor NF-E2-related factor 2 (Nrf2) is a key protective mechanism of pancreatic β-cells against oxidative stress under diabetic conditions, as its activation leads to the expression of antioxidant genes [124]. In vivo, Nrf2 reduces ROS levels and decreases the β-cell apoptosis mediated by NO [125]. Conversely, the transcriptional factor NF-κB, leads to an increase in ROS levels. This ROS production is activated by the proinflammatory IL-1β signalling pathway, which also controls insulin production in vitro and in murine islets [126]. Cytokines secreted from endothelial cells subjected to oxidative stress could activate this signalling pathway in pancreatic β-cells, leading to a positive feedback loop of inflammation and oxidative stress.

The enzymatic XO system is closely related with the diabetic disease and the dysfunction of pancreatic β-cells. In patients with T2D, XO activity observed in the serum directly correlates with IR [127] and the glucose levels in overweight women [128]. In vivo, there is some discussion about the role of XO in streptozotocin-induced T1D model. Some authors indicate that streptozotocin induces ROS in pancreatic β-cells through XO, which reduces cell viability and insulin production [129, 130]. However, other reports indicate that the pathological changes caused in pancreatic β-cells by XO are different than those caused by streptozotocin [131]. In diabetic rats induced by streptozotocin, BAC (4-methyl-2-[(2-methylbenzyl) amino]-1,3-thiazole-5-carboxylic acid), a XO inhibitor, regulates glucose levels and improves the responsiveness to insulin. This treatment also restores antioxidant levels and decreases the level of inflammatory markers [132]. In vitro, INS-1 cells exposed to intermittent high glucose levels show an increased expression of XO [133]. Finally, the supplementation of isolated pancreatic islets with XO temporarily increases insulin release [134]. The activity of XO, induced or not by streptozotocin, could impact on endothelial cells surrounding the pancreatic β-cells, promoting immune cell infiltration and trigger an autoimmune response.

It is known that human pancreatic β-cells are more resistant to NO levels than murine pancreatic β-cells in vitro. This protection is mainly mediated by heat shock protein 70 (hsp70) [135]. Different groups have reported beneficial and pathological effects for this molecule. NO protects against the mitochondrial-derived oxidative stress in the response to EMCV virus, which specifically damages MIN6 cells [136]. Nevertheless, NO also leads to apoptosis in mouse insulinoma MIN6 cells, with the endoplasmic reticulum playing a key role [137]. Remarkably, the endoplasmic appears to underlie NO-mediated protective effects in the same cell line and cultured murine islets [138]. It is noteworthy, that endothelial cells produce and release NO physiologically, which can be a potential source of oxidative stress and affect β-cells. However, this paracrine signalling pathway remains unexplored as these results are only derived from in vitro and ex vivo studies.

More recently, a strong relationship between ROS derived from NOX activity and pancreatic β-cells has been described. NOX activity plays a key role in the dysfunction of INS-1 cells and human β-cells (1.1B4) induced by glucotoxicity and CD36 [139]. More specifically, in murine pancreatic islets and Ins-1 cells, NOX1 is activated by TNF-α, IL-1β and IFN-ϒ, which increases ROS and leads to apoptosis. NOX1 activation seems to impair GSIS, which is preserved when cells are incubated with the specific inhibitor ML171 [140]. Again, endothelial cells could be the source of the proinflammatory cytokines released to the pancreatic environment, which can activate NOX1 at pancreatic β-cells.

In 2003, NOX2 was described for the first time to be expressed in pancreatic β-cells [141]. Later, NOX2 was found to be expressed in insulin granules, where it negatively regulates insulin production in response to glucose in murine islets by the cAMP/PKA pathway [142]. In a NOX2 *knock-out* mouse model injected with streptozotocin, mice had lower plasma glucose levels, as well as increased insulin secretion and glucose tolerance. In this model, NOX2 absence also reduces apoptotic levels and insulitis [143]. Similar findings regarding the pathological effects of NOX2 are observed when cells are exposed to very-low-density lipoproteins instead of high glucose levels [144]. Although other authors confirm the role of NOX2 in insulin secretion also demonstrate that NOX2 *knock-out* pancreatic islets are not protected from glucotoxicity and present similar apoptosis [145].

NOX4 is expressed in human pancreatic islets [146]. NOX4 also plays a physiological role in GSIS by the production of H_2_O_2_. In fact, the β-cell-specific NOX4 *knock-out* mouse model presents glucose intolerance and a peripheral impaired response to insulin [147]. Some authors indicate that NOX4 inhibitors reduce ROS production in response to high glucose and palmitate, and in response to proinflammatory cytokines. These data demonstrate a pathological role for NOX4 in pancreatic β-cells [146, 148].

Finally, NOX5 is also expressed in human pancreatic islets. Specifically, its expression is induced in β-cells in response to high glucose levels. Nonetheless, as NOX5 is absent in the rodent genome its study is more limited. In the β-cell-specific NOX5 *knock-in* mouse model, the GSIS is reduced after 14 weeks of high-fat diet. Similarly, NOX5-expressing murine islets present an impaired insulin secretion when stimulated with palmitate [149]. However, there is paucity of information regarding NOX3, DUOX-1 and DUOX-2 expression in pancreatic β-cells.

In summary, ROS play a physiological role in pancreatic β-cells. More importantly, ROS derived from different NOX isoforms seem to regulate and/or participate in physiological insulin secretion [142, 147]. Nevertheless, excessive activation of ROS sources leads to pancreatic β-cell dysfunction and impaired GSIS, in vitro, in vivo, and in human and murine pancreatic islets [140, 148, 149]. Endothelial cells could potentially induce oxidative stress in pancreatic β-cells by different mechanisms. Firstly, they could be a source of locally produced ROS. Secondly, they regulate the amount of glucose reaching pancreatic β-cells, the impairment of this mechanism could produce intermittent high levels of glucose, leading to oxidative stress in pancreatic β-cells. Finally, cytokines released by endothelial cells as IL-1β could impact signalling pathways that induce ROS production in pancreatic β-cells.

### Oxidative stress affects the endothelial–β cell paracrine environment

In the context of human pathophysiology, the effect of oxidative stress is not limited to a specific cell type, it also exerts a paracrine effect in the proximal cells. In the case of the Langerhans islets, oxidative stress can affect any endocrine cell and the endothelial cells influencing them. For instance, the NO produced by endothelial cells induces insulin secretion of pancreatic β-cells [80]. In this section, we will focus more specifically on how oxidative stress can influence the communication between endothelial and pancreatic β-cells.

As mentioned before, VEGF-A is key in the vascularization of the endocrine pancreas and the contact between pancreatic β-cells and endothelial cells [72–75]. Interestingly, VEGF expression is influenced by oxidative stress in different cell types. The preincubation of MIN6 cells with N-acetylcysteine prevents VEGF production induced by palmitate stimulation [150]. JunD is a transcription factor that protects from oxidative stress. JunD *knock-out* mice present increased oxidative stress in the pancreatic islets, as well as higher VEGF expression and vascularization [151]. Interestingly, oxidative stress also regulates VEGF secretion in diabetic retinopathy. MIO-M1 retinal cells *and ex vivo* retinal explants increase VEGF expression in response to oxidative stress via HIF-1α [152]. Similar findings have been observed in retinal pigment epithelial (ARPE19) cells [153]. This oxidative stress-induced regulation of VEGF is also present in macrophages [154]. Therefore, there is a well-stablished crosstalk between ROS levels and VEGF-A, the main factor regulating the signalling between endothelial and β-cells. The development of the diabetic disease is accompanied by increasing ROS, which will then affect the levels of VEGF-A secretion and its different effects on β-cell homeostasis.

Other soluble factors that mediate the interactions between endothelial and pancreatic β-cells are T-cadherin, Serpin E1 and HB-EGF [82, 84, 87]. These molecules also have a strong relationship to oxidative stress. T-cadherin protects HUVEC from apoptosis by activating the mTOR survival signalling pathway [155]. Serpin E1 or plasminogen activator inhibitor 1 is associated with cardiovascular and metabolic diseases. The expression of this factor is regulated by both oxidative stress and hypoxia [156]. Moreover, there is some discussion about the relationship between HB-EGF and oxidative stress. On the one hand, HB-EGF exerts antioxidant effects in some cells, as stromal cells. HB-EGF increases the action of catalase, SOD and GPX enzymes [157]. On the other hand, H_2_O_2_ supplementation increases HB-EGF expression in epithelial gastric cells [158]. In T1D paediatric patients, HB-EGF plasma levels are increased, as well as markers of inflammation and oxidative stress [159].

Apart from the alteration of these soluble factors, oxidative stress can lead to other major pathological mechanisms involving endothelial and pancreatic β-cells: inflammation. It has been discussed how oxidative stress can increase the production of different cytokines and proinflammatory mediators as IL-6, PGE_2_ and MCP-1 in endothelial cells [119–123]. These soluble factors can have a paracrine effect on pancreatic β-cells in the Langerhans islet.

IL-6 seems to play a protective role in pancreatic β-cells. IL-6 promotes phagocytosis in Ins-1 cells, protecting them from stress and reducing apoptotic levels [160]. In similar models, IL-6 protects from the action of other proinflammatory cytokines, as it increases the viability of β-cells [161]. In vivo, the overproduction of IL-6 increases GSIS in mice, improving glucose tolerance. This has an in vitro and ex vivo correlate: the preincubation of MIN6 cells and pancreatic islets with IL-6 increases GSIS [162]. Additionally, PGE_2_ secreted from endothelial cells may also have a paracrine effect. In diabetic patients, the EP_3_ receptor (activated by PGE_2_) is overexpressed in pancreatic islets. Some reports indicate that PGE_2_ exhibits an inhibitory effect on GSIS in pancreatic β-cells [163, 164]. Finally, although β-cells do not express the specific receptor for MCP-1, it can activate the proinflammatory signalling cascade of NF-κB via Gi-coupled receptors in MIN6 cells and pancreatic islets [165]. This paracrine signalling can also act in the opposite direction. Pancreatic β-cells release TNF-α in response to oxidative stress, which can have a paracrine impact on endothelial cells [104, 117, 140]. The stimulation of endothelial cells with TNF-α leads not only to an increased immune cell infiltrate but also to a reduction in the insulin response [166]. All these described signalling pathways show how oxidative stress could contribute to the imbalance between protective (IL-6) and detrimental (PGE_2_, MCP-1, TNF-α) cytokines. This imbalance would potentially lead to a proinflammatory loop and therefore extend the progression of β-cell dysfunction.

Oxidative stress leads to an increased expression of ICAM-1 or VCAM-1 in endothelial cells, which facilitates immune infiltration [115, 117]. In the specific case of the pancreatic islets, this likely promotes the infiltration of immune cells and accelerate the progression of both T1D and T2D. This could be even more aggravated by the different cytokines secreted by both cell types to the paracrine environment.

**Fig. 2:**
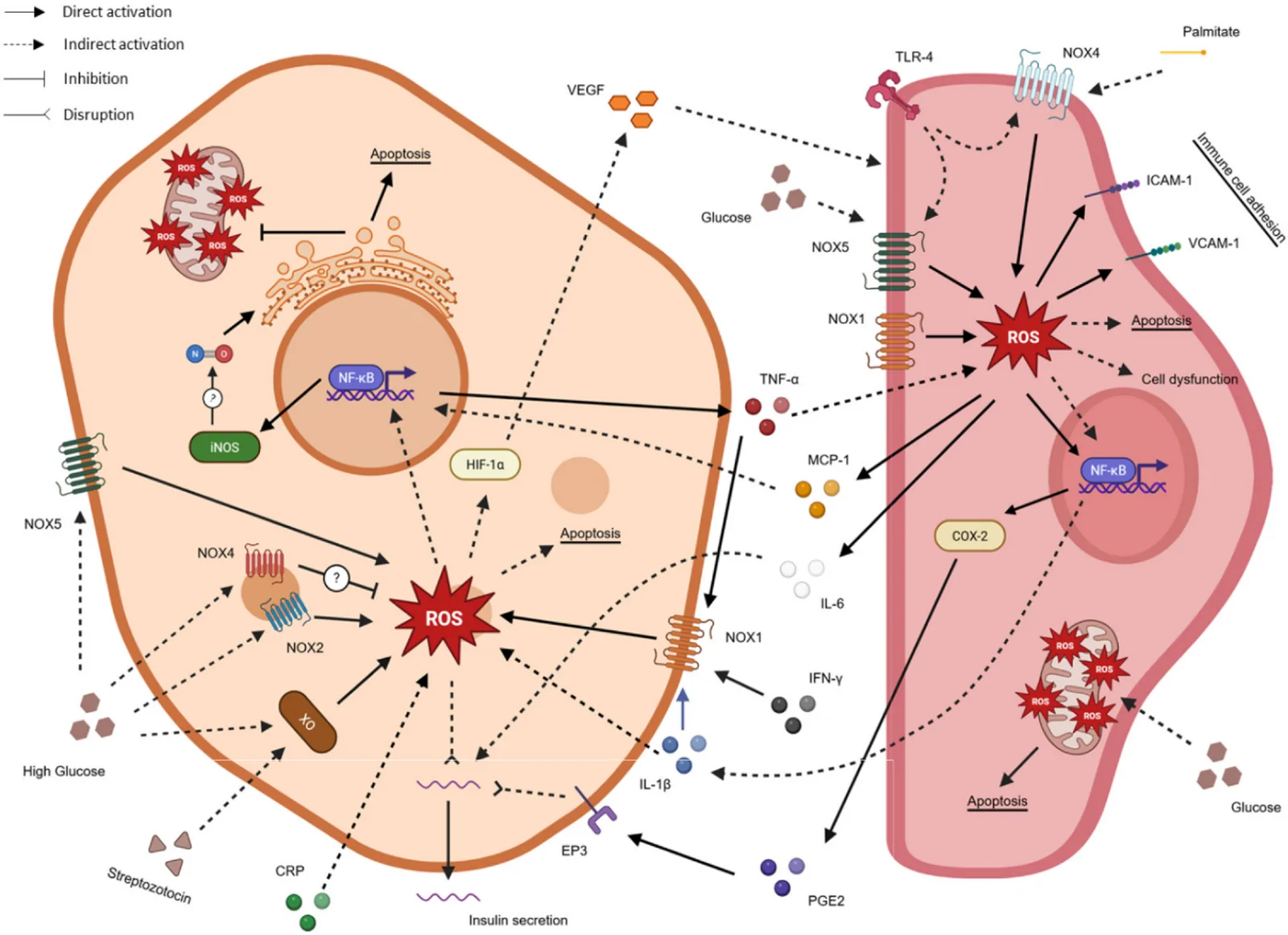
Oxidative stress promotes a pro-inflammatory microenvironment that disrupts pancreatic β-cells, endothelial cells, and their paracrine crosstalk, establishing a potential positive feedback loop. Under pro-inflammatory/diabetic conditions, reactive oxygen species (ROS) are generated in both cell types. *In pancreatic β-cells*, glucose stimulates NOX2, NOX4, NOX5 and xanthine oxidase (XO) activity. Excess ROS derived from these enzymes *impair insulin secretion* and *trigger apoptosis*, as well as *NF-κB* and *HIF-1α* signalling. NF-κB activation promotes TNF-α secretion, which could further enhance ROS production in both β-cells and endothelial cells. HIF-1α activation increases VEGF secretion, potentially affecting endothelial cell function. *In endothelial cells*, glucose activates NOX5 and mitochondrial ROS production, while palmitate stimulates NOX4. Both NOX4 and NOX5 are also activated via TLR4-mediated inflammatory signalling. Oxidative stress in endothelial cells induces *NF-κB* activation, *inflammation*, *dysfunction*, and *apoptosis*. Endothelial NF-κB activation increases PGE_2_ and IL-1β production, which could inhibit insulin secretion and stimulate ROS generation in β-cells, respectively. ROS also promote IL-6 secretion, potentially enhancing insulin synthesis, and MCP-1 secretion, which might activate NF-κB in β-cells. CRP: C-reactive protein. EP_3_: PGE_2_ receptor 3. HIF-1α: hypoxia inducible factor 1α. ICAM-1: intracellular cell adhesion molecule 1. IFN-γ: interferon γ. IL-1β: interleukin 1β. IL-6: interleukin 6. iNOS: inducible nitric oxide synthase. MCP-1: monocyte chemoattractant protein 1. NF-κB: Nuclear factor kappa B. NO: nitric oxide. NOX: NADPH oxidase. PGE_2_: prostaglandin E_2_. TLR-4: toll-like receptor 4. TNF-α: tumoral necrosis factor α. VCAM-1: vascular cell adhesion molecule 1. VEGF: vascular endothelial growth factor. XO: xanthine oxidase. (◊): direct activation. (): indirect activation. (**-I**): inhibition. (**--<**): disruption. Figure created using Biorender

In summary, there is a potential relationship between oxidative stress and the paracrine signalling between endothelial cells and pancreatic β-cells that needs to be better addressed. Firstly, it has been described how oxidative stress can affect VEGF expression and production, which is the main mediator in this paracrine communication. This relationship was demonstrated in vitro, in vivo and ex vivo by different research groups. Secondly, the information about how oxidative stress affects the other paracrine signalling pathways or mediators such as T-cadherin, Serpin E1 or HB-EGF is scarce and mainly derived from in vitro data. Thirdly, although there is interesting data about the role of oxidative stress-derived inflammation in the paracrine signalling, this information is derived from in vitro and ex vivo studies, indicating the need of more human and in vivo studies. The proposed studies on oxidative stress as a therapeutic target could help identify ways to reduce complications associated with diabetes (Fig. 2).

## Conclusion and therapeutic perspective

The paracrine signalling between endothelial and pancreatic β-cells is essential for the development and physiology of the islets of Langerhans. Under diabetic conditions, this communication becomes impaired, largely driven by oxidative stress. Oxidative stress affects both endothelial cells and β-cells, as well the crosstalk through two interconnected mechanisms. First, it promotes proinflammatory cytokine production, which not only affects the function of the other cell type but also recruits inflammatory cells. Second, it compromises the GSIS of pancreatic β-cells, an effect further exacerbated by the proinflammatory environment. Together, these processes lead to dysfunction of both cell types and a positive proinflammatory feedback loop that worsens the diabetic situation. Although interesting, most of these observations need to be confirmed in human and in vivo studies, as most of the information is obtained from in vitro studies. Once the key signalling pathways of the endothelial–β-cell paracrine signalling and their ROS disruptors are identified, a promising yet underexplored therapeutic strategy will arise: to target ROS sources that disrupt endothelial–β-cell paracrine signalling.

Advocating antioxidant therapy interferes with the physiological ROS signalling in the organism. In fact, antioxidant therapies as N-acetylcysteine have failed in their application to different diseases due to the lack of specificity. These therapies are targeted to block ROS levels, but not their enzymatic source. The development of novel inhibitors specific for enzymatic sources, as the different NOX isoforms, could be key improving the specificity [167]. In this regard, it has been observed how different NOX isoforms may play a different role. Interestingly, while NOX5 is associated with β-cell dysfunction under diabetic conditions [122, 149], there is controversy around the pathophysiological roles played by NOX2 [142–145] and NOX4 [146–148], which indicates the need of these isoform-specific inhibitors. These isoform-specific inhibitors not only would help to better characterize the effect of each enzyme in this paracrine signalling but also would serve as potential therapies to reestablish this impaired communication in diabetes. Moreover, the vehiculation of these drugs to the pancreatic tissue would be another tool to overcome the problems of the antioxidant therapy. In fact, different technologies have been developed and tested in the last years to improve the specificity and availability of the therapeutic molecules in the pancreatic tissue. These technologies include the use of designed exosomes, or functionalised nanoparticles containing antibodies against β-cell specific antigens [168, 169]. Collectively, an improvement of the specific inhibitors against ROS producing enzymes together with a targeted delivery, could make the reestablishment of the homeostatic endothelial–β-cell paracrine signalling a promising therapeutic approach.

## Abbreviations

DM: Diabetes mellitus
DUOX: Dual oxidase
GLP-1: Glucagon like peptide 1
GLUT-2: Glucose transporter 2
GSIS: Glucose-stimulated insulin secretion
HB-EGF: Heparin-binding epidermal growth factor
HGF: Hepatocyte growth factor
HIF-1α: Hypoxia inducible factor 1α
HLA: Human leukocyte antigen
H_2_O_2_: Hydrogen peroxide
HO^•^: Hydroxyl radical
HUVEC: Human umbilical vein endothelial cells
ICAM-1: Intracellular adhesion molecule 1
IFN-γ: Interferonγ
IL-1β: Interleukin 1 beta
IL-6: Interleukin 6
IR: Insulin resistance
MCP-1: Monocyte chemoattractant protein 1
NF-κB: Nuclear factor kappa B
NO: Nitric oxide
NOS: Nitric oxide synthase
NOX: NADPH oxidase
Nrf2: NF-E2-related factor 2
O_2_: Molecular oxygen
O_2_^•-^: Superoxide anion
ONOO^−^: Peroxynitrite
Pdx-1: Pancreas/duodenum homeobox 1
PGE_2_: Prostaglandin E_2_
ROS: Reactive oxygen species
SGLT2: Sodium-glucose cotransporter 2
SOD: Superoxide dismutase
T1D: Type I diabetes
T2D: Type II diabetes
TLR4: Toll-like receptor 4
TNF-α: Tumoral necrosis factor alpha
VCAM-1: Vascular cell adhesion molecule 1
VEGF: Vascular endothelial growth factor
XO: Xanthine oxidase
